# Supplementary material for: GAN-based medical image small region forgery detection via a two-stage cascade framework
Source: PLoS One. 2024 Jan 2;19(1):e0290303. doi: 10.1371/journal.pone.0290303 (PMC10760893; doi:10.1371/journal.pone.0290303)
Supplement: Attachment — Submitted filename: reviewer comments - Mai-22022023.docx [file pone.0290303.s001.docx]

**Reviewer Comments**

**Dear Editor(s), February 22, 2023**

I am pleased to submit my comments for the submitted manuscript (**PONE-D-23-02950**) entitled “**GAN-based Medical Image Small Region Forgery Detection via a Two-Stage Cascade Framework**” to be taken into consideration for publication by your esteemed journal “***PLOS ONE***”.

Due to the privacy of medical images, the lack of data has always been a significant problem for machine learning tasks related to medical images. One way to solve this problem is the generative adversarial network (GAN), which can generate images that are highly similar to real images. Using GAN for data enhancement of medical images is significantly helpful for many computer-aided diagnosis (CAD) tasks. The image quality generated by GAN is enough to confuse radiologists. A new GAN-based automated tampering attack, like CT-GAN, has emerged. It can inject or remove lung cancer lesions to CT scans. Therefore, CT-GAN can make doctors misjudge the patient’s condition, seriously threatening the patient’s life safety. However, new attacks like CT-GAN challenge the current detection methods. This type of attack is a GAN-based automated 3D tampering attack. It generates only a minimal area, and the surrounding area is used as a constraint condition to train a conditional generative adversarial network (CGAN). Because the tampering region may even account for less than 1% of the original image, even state-of-the-art methods are challenging to detect the traces of such tampering. At present, no solution can effectively detect the GAN-based small region forgery attack in medical images.

This paper has successfully proposed a novel two-stage cascade framework based on a local detection network and a global classification method that can detect GAN-based small region forgery attacks in medical images. In the local detection stage, the detector network was trained with small sub-images so that interference information in authentic regions will not affect the detector. Then, depthwise separable convolution and residual networks were used to prevent the detector from over-fitting and enhance the ability to find forged regions through the attention mechanism. After training, the detector can detect the tampered region effectively. The detection results of all sub-images in the same image will be combined into a heatmap. In the global classification stage, using gray-level co-occurrence matrix (GLCM) can better extract features of the heatmap. This novel cascade framework can successfully classify whether a CT image has been tampered and locate the tampered position. Compared with the method that uses the whole image as input, this method can locate the tampered coordinate and requires less training data but has faster training speed and higher accuracy. Sufficient experiments show that this method can achieve excellent performance than the state-of-the-art detection methods. To the knowledge of this paper, there is no detection method for GAN forged medical images. Although there is no specific solution to detect the medical images generated by GAN, there are some domain generic methods.

The authors have done good work on the title “**GAN-based Medical Image Small Region Forgery Detection via a Two-Stage Cascade Framework**”. It will add new knowledge and new areas of research to the subject area compared with other published material.

**However, i have some minor concerns:**

1. The authors have inserted Fig. 1 in the introduction section, but it was not cited inside the manuscript. Kindly check the guideline of the PLOS ONE journal and perform the required amendment.
2. It would be more appropriate for the authors to define abbreviations upon first appearance in the main text such as PCA, GAN, CAD, GLCM, CCPA, GDPR and CV field; principal component analysis (PCA), generative adversarial network (GAN) computer-aided diagnosis (CAD), gray-level co-occurrence matrix (GLCM), California consumer privacy act (CCPA) and general data protection regulation (GDPR) and computer vision (CV), respectively.
3. In the section of “Challenges”, it would be more appropriate for the authors to inserted Fig. 4 immediately after the paragraph in which the figure is cited.
4. In the following sentence “That is why even state-of-the-art methods are challenging to detect the tampering trace of CT-GAN. Because of this, many methods based on statistical characteristics, such as [6, 10, 11]”, it would be more appropriate for the authors to detail the methods with their related citation.
5. In the section of “Local detection network architecture”, it would be more appropriate for the authors to inserted Fig. 5 immediately after the paragraph in which the figure is cited.
6. In the section of “Detect CT scans”, it would be more appropriate for the authors to inserted Fig. 8 immediately after the paragraph in which the figure is cited.
7. The following sentence “When the forgery attack is applied to the whole image, although we notice that our method can also detect the tampered region with high accuracy, the efficiency still cannot catch up with those methods that treat the target as a single unit, such as [7]”, seemed as incomplete sentence. Kindly check it and perform the required amendment.
8. In the following sentence “The works such as [29–34] are based on the CycleGAN structure”, it would be more appropriate for the authors to briefly indicate the works with their related citation.
9. In section of “Evaluation “, the following sentence “Similarly, for a real region, suppose 9 or more consecutive slices in the real slice are judged as positive examples, or 9 of the 10 consecutive slices are judged as positive examples”, seemed as redundant and hanged sentence. Kindly check it and perform the required amendment.
10. **Moderate editing is required throughout the manuscript, for example:**
a. “The design of residual block refers to ResNet [39]. The number of convolution kernels channels is unchanged when the input feature image size is the same as the output”. Moderate editing is required.
b. “At the end of our network. Selu [47] is used as the activation function in the full connection layer of the network”. Moderate editing is required.
c. “The crop operation is because the practical part of the CT image is only the inside of a circle tangent to the square frame. Moderate editing is required
d. “s. Therefore, after local detection of the tampered slice, a series of sub-images will be judged as positive in theory. Therefore, the tampering trace of CT-GAN can be detected through some fixed modes, such as the vertical and horizontal continuous n sub-images are judged to be false”. Moderate editing is required.
e. “However, this detection method is not perfect. The size of the tampered region of GAN is not fixed. The attacker can set the size of the CT-GAN tampered region to a certain extent”. Moderate editing is required.

Best regards,

Dr. Mai Abdel Haleem Abusalah

Faculty of Medical Allied Science,

Zarqa University,

Zarqa, 13110, Jordan.

Tel: +962-796862347

e-mail: [ellamomo88@yahoo.com](mailto:ellamomo88@yahoo.com)
